# α-Synuclein A53T Binds to Transcriptional Adapter 2-Alpha and Blocks Histone H3 Acetylation

**DOI:** 10.3390/ijms22105392

**Published:** 2021-05-20

**Authors:** Ji-Yeong Lee, Hanna Kim, Areum Jo, Rin Khang, Chi-Hu Park, Soo-Jeong Park, Eunsang Kwag, Joo-Ho Shin

**Affiliations:** 1 Department of Pharmacology, Sungkyunkwan University School of Medicine, Suwon 16419, Korea; ljyljy915@naver.com (J.-Y.L.); neuro_brain@naver.com (H.K.); alm7760@gmail.com (A.J.); rkhang777@gmail.com (R.K.); soojung8725@naver.com (S.-J.P.); kes412@nate.com (E.K.); 2 Single Cell Network Research Center, Sungkyunkwan University School of Medicine, Suwon 16419, Korea; 3 YEPBIO Co., Ltd., Anyang 14056, Korea; chihupark@naver.com; 4 Samsung Medical Center, Samsung Biomedical Research Institute, Seoul 06351, Korea

**Keywords:** α-synuclein, Parkinson’s disease, transcriptional adapter 2-alpha, histone acetylation, neurotoxicity

## Abstract

α-Synuclein (α-syn) is a hallmark amyloidogenic protein component of Lewy bodies in dopaminergic neurons affected by Parkinson’s disease (PD). Despite the multi-faceted gene regulation of α-syn in the nucleus, the mechanism underlying α-syn crosstalk in chromatin remodeling in PD pathogenesis remains elusive. Here, we identified transcriptional adapter 2-alpha (TADA2a) as a novel binding partner of α-syn using the BioID system. TADA2a is a component of the p300/CBP-associated factor and is related to histone H3/H4 acetylation. We found that α-syn A53T was more preferentially localized in the nucleus than the α-syn wild-type (WT), leading to a stronger disturbance of TADA2a. Indeed, α-syn A53T significantly reduced the level of histone H3 acetylation in SH-SY5Y cells; its reduction was also evident in the striatum (STR) and substantia nigra (SN) of mice that were stereotaxically injected with α-syn preformed fibrils (PFFs). Interestingly, α-syn PFF injection resulted in a decrease in TADA2a in the STR and SN of α-syn PFF-injected mice. Furthermore, the levels of TADA2a and acetylated histone H3 were significantly decreased in the SN of patients with PD. Therefore, histone modification through α-syn A53T-TADA2a interaction may be associated with α-syn-mediated neurotoxicity in PD pathology.

## 1. Introduction

Parkinson’s disease (PD) is the second most common progressive neurodegenerative disorder, characterized phenotypically by motor abnormalities such as bradykinesia, rigidity, tremor, and neuropsychiatric disturbances resulting from selective dopaminergic neuronal death in the substantia nigra (SN) [1].

Although the majority of PD cases are sporadic or have no known causes, there are several autosomal dominant or recessive familial PD cases linked to genetic factors [2]. To date, more than 16 PD-associated loci have been identified, with *α-synuclein* (*α-syn*) being one of the most extensively studied PD genes [3]. There are emerging mechanisms that have been shown to underlie α-syn-associated toxicity. First, there are point mutations in α-syn, including A53T, A30P, and E46K, that are linked to familial PD. Among these, α-syn A53T has a high tendency to form α-syn protofibril [4]. Cell-to-cell propagation of misfolded α-syn is also considered a key toxic mechanism underlying α-syn pathology. In addition, the accumulation of α-syn along the endoplasmic reticulum (ER) membrane triggers ER stress by interfering with intracellular trafficking and vesicle transport [2]. α-Syn accumulation at synaptic sites is considered the main cause of neuronal death in PD [5] and its mitochondrial localization in human dopaminergic neurons increases the production of reactive oxygen species (ROS) [6]. Interestingly, the nuclear localization of α-syn was studied in several papers for decades. However, the effect of the nuclear localization of α-syn is still under investigation [7,8,9,10,11,12]. For example, α-syn can translocate to the nucleus in mice injected with paraquat, an herbicide [8]. In addition, the nuclear-localization-sequence (NLS)-tagged α-syn increases neurotoxicity when it is overexpressed in SH-SY5Y cells and flies [9]. Furthermore, the formation of nuclear α-syn inclusions was identified in patients with multiple system atrophy, a well-known sporadic synucleinopathy [10,11].

In this study, we identified a novel interactome of α-syn wild-type (WT) and A53T, to help to expand our knowledge on the role of nuclear α-syn in neurotoxicity. To identify a physiological binding partner of α-syn, we applied the BioID system, which is based on the fusion of a promiscuous *Escherichia coli* biotin ligase to α-syn for proximity-dependent labeling of interacting proteins in living cells [13]. BioID can detect weak or transient interactions that occur in vivo for a defined period and is also applicable to insoluble proteins [14].

Herein, we identified transcriptional adapter 2-alpha (TADA2a) as a novel binding partner of α-syn and found that α-syn A53T strongly blocked TADA2a activity, leading to reduced histone H3 acetylation. These results were confirmed using a mouse model of PD and in patients with PD.

## 2. Result

### 2.1. BioID-Identified Binding Partners of α-Syn

**Figure 1:**
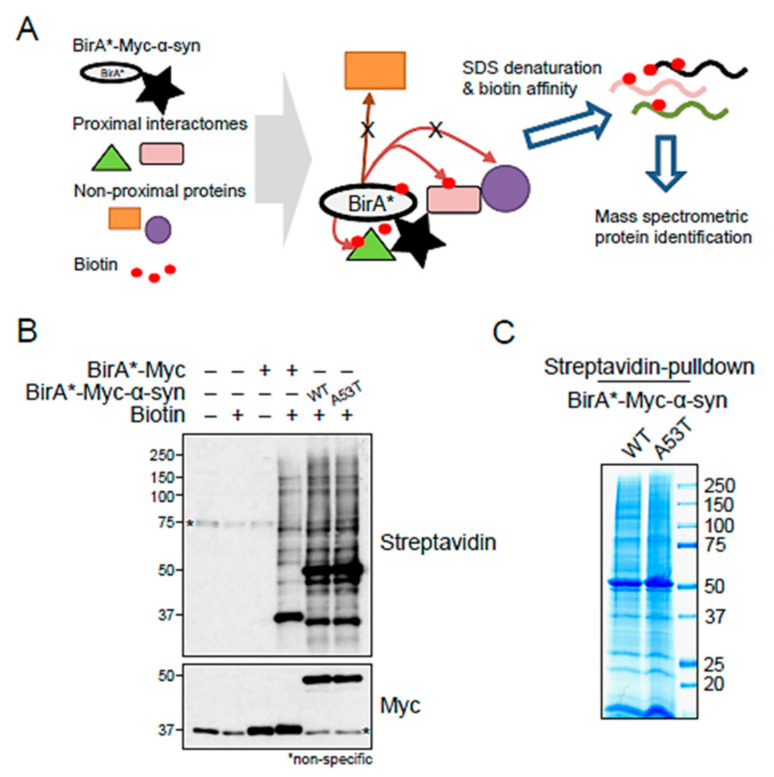
BioID-identified binding partners of α-syn. (**A**) Schematic image of BioID pulldown assay with BirA*-fused, Myc-tagged α-syn. (**B**) Biotinylated proteins were isolated and detected using a streptavidin antibody. * Non-specific band. (**C**) Representative image of biotin-labeled immunoprecipitates of BirA*-fused, Myc-tagged α-syn WT and A53T visualized using Coomassie staining.

The BioID system is the most widely used proximity labeling method and features a mutant biotin ligase BirA* (Arg118Gly) fused to the protein of interest (Figure 1A) [13,15]. BirA*-fused, Myc-tagged control, α-syn WT, and α-syn A53T were transfected into SH-SY5Y cells in the presence of biotin. Biotinylated proteins were isolated via streptavidin pulldown and detected using a streptavidin antibody (Figure 1B), showing that the BirA* domain successfully labeled BirA* and other diverse proteins. To identify the biotin-labeled proteins in α-syn WT and A53T, biotin-labeled immunoprecipitates were separated using SDS-PAGE and stained with Coomassie blue for mass spectrometric analysis (Figure 1C).

### 2.2. Proteomic Analysis of α-Syn WT and A53T Interactors

**Figure 2:**
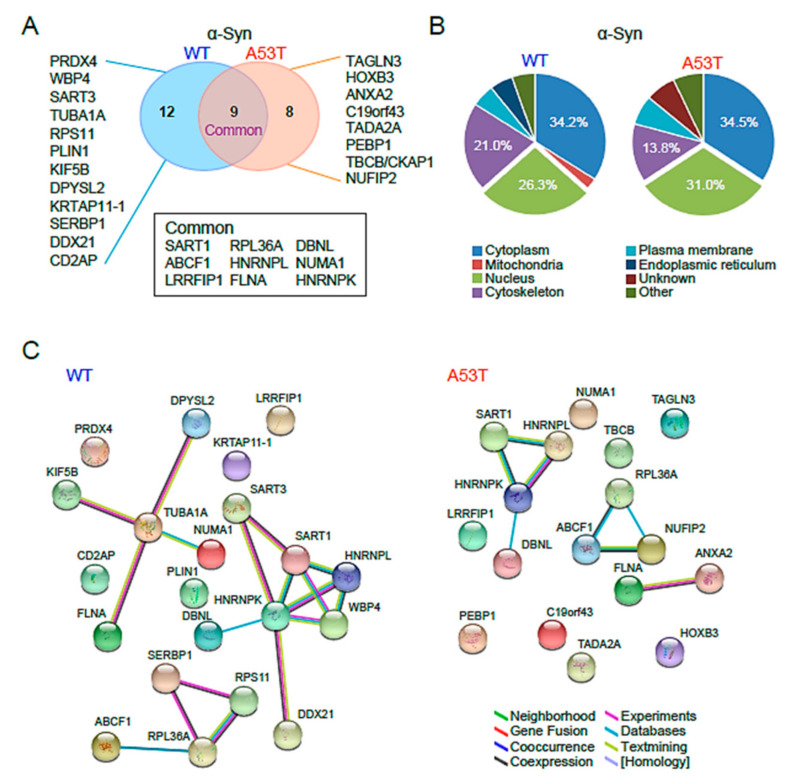
Proteomic analyses of α-syn WT and A53T interactors. (**A**) α-Syn WT and A53T interactors were identified via mass spectrometry following the BioID pulldown assay. (**B**) Pie chart showing the physiological relevance of α-syn interactors. (**C**) Proteins interacting with α-syn WT or A53T were analyzed using functional clustering. Biological pathway clusters enriched in the proteome of differentially expressed genes are shown. Line color annotation: green, neighborhood; red, gene fusion; blue, co-occurrence; black, co-expression; pink, experiments; light blue, databases; light green, text mining; light purple, homology.

The mass spectrometric analysis identified 34 biotin-labeled proteins in BirA*-fused, Myc-tagged α-syn WT overexpression, and 22 proteins in BirA*-fused, Myc-tagged α-syn A53T overexpression. Thirteen of the thirty-four WT-interacting proteins and five of the twenty-two A53T-interacting proteins were reported in different BioID settings [13], suggesting that those repeatedly identified in different studies may be false-positive interactors. Since nine proteins were commonly identified both in α-syn WT and A53T interactors, we assigned twelve and eight proteins as α-syn WT and A53T interactors, respectively (Figure 2A, Supplementary Table S1).

A Software Tool for Rapid Annotation of Proteins (STRAP) analysis was used to deduce the physiological relevance of α-syn interactors (Figure 2B). Graphical representation of gene ontology (GO)-associated categories are displayed in terms of cellular components and molecular functions (Figure 2B). Interactors of both α-syn WT and A53T were largely present in the cytoplasm and nucleus. However, the nucleus had a larger proportion of α-syn A53T than α-syn WT, while the cytoplasm had comparable proportions of α-syn A53T and WT, suggesting that α-syn A53T may play an important role in synucleinopathies in the nucleus.

Analysis with the STRING software showed that many α-syn WT-interacting partners were grouped into structural proteins, translational machinery, and RNA-binding proteins with functional associations, including neighborhood (green), gene fusion (red), co-occurrence (blue), co-expression (black), experimental (pink), database (light blue), and text mining (light green). However, α-syn A53T-interacting proteins were weakly classified, suggesting that α-syn A53T behaved differently than α-syn WT (Figure 2C).

### 2.3. α-Syn A53T Is Located in the Nucleus and Binds Strongly to TADA2a

The direct effect of α-syn on transcription in the nucleus has been demonstrated and α-syn A53T differently regulates gene expression compared with α-syn WT [16]. Although a functional relationship between α-syn and histone acetylation has been emerged [8,9], there was a missing puzzle piece to understand the mechanism underlying the modulation of histone acetylation by α-syn. Herein, we selected TADA2a, a component of the histone acetyltransferase (HAT) complex, to understand how α-syn regulates gene expression in PD pathogenesis [17].

**Figure 3:**
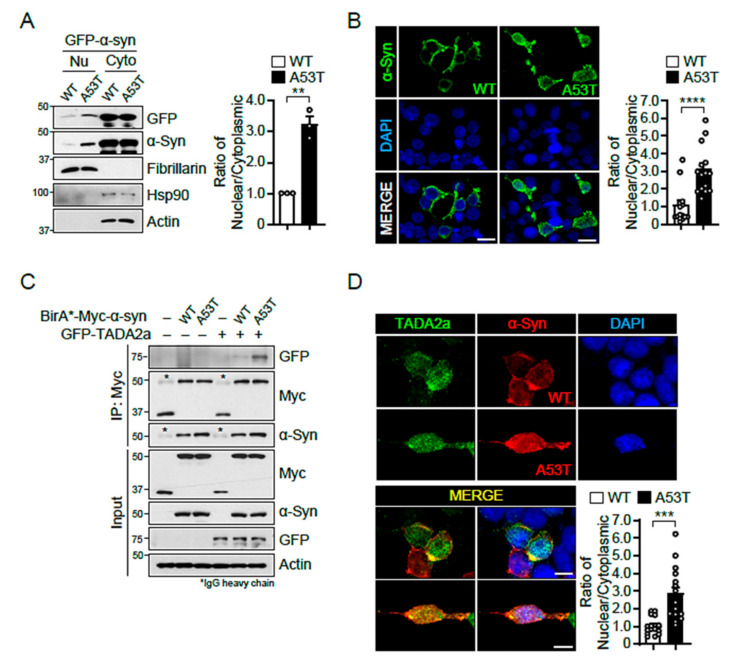
α-Syn A53T is located in the nucleus and binds strongly to TADA2a. (**A**) Nuclear and cytoplasmic fractions of GFP-tagged α-syn WT and A53T transfected SH-SY5Y cells were separated via SDS-PAGE and probed for GFP and α-syn. The cytoplasmic marker Hsp90 appeared in the cytoplasmic fraction whereas the nuclear marker fibrillarin appeared in the nuclear fraction. Quantification of the ratio of nuclear/cytoplasmic GFP-tagged α-syn WT and A53T from immunoblot analysis, *n* = 3 per group (right panel). (**B**) Immunofluorescence images of Flag-tagged α-syn WT or A53T (green), DAPI (blue), and merged in transfected SH-SY5Y cells. These images show the differential localization between α-syn WT and A53T. Scale bars = 20 μm. Quantification of the ratio of nuclear/cytoplasmic Flag-tagged α-syn WT or A53T from immunofluorescence images, *n* = 15 cells per group (right panel). (**C**) Immunoprecipitated BirA*-fused, Myc-tagged α-syn A53T interacts with GFP-tagged TADA2a more strongly than α-syn WT. * IgG heavy chain band. (**D**) Immunofluorescence images of GFP-tagged TADA2a (green), Flag-tagged α-syn WT/A53T (red), DAPI (blue), and merged in transfected SH-SY5Y cells. The merged images show that α-syn A53T co-localized with TADA2a in the nucleus. Scale bars = 10 μm. Quantification of the ratio of nuclear/cytoplasmic Flag-tagged α-syn WT or A53T from immunofluorescence images, *n* = 15 cells per group (right bottom panel). Data = mean ± SEM. Statistical significance was determined using an unpaired, 2-tailed Student’s *t*-test (A, B, and D). Differences were considered significant at *p* < 0.05. ** *p* < 0.01, *** *p* < 0.001, and **** *p* < 0.0001.

To validate whether α-syn A53T is localized in the nucleus, GFP-tagged α-syn WT and A53T were transfected into SH-SY5Y cells and their localizations were assessed via subcellular fractionation (Figure 3A). Hsp90 and fibrillarin were used as the subcellular markers for cytoplasm and nuclear fraction, respectively. Results showed that the ratio of nuclear/cytoplasmic localization was higher for α-syn A53T than for α-syn WT. Fluorescence images of Flag-tagged α-syn WT and A53T also confirmed the nuclear localization of α-syn A53T (Figure 3B). Next, we performed a co-immunoprecipitation assay to assess the interaction between α-syn and TADA2a in SH-SY5Y cells transfected with BirA*-fused, Myc-tagged α-syn WT and A53T, and GFP-tagged TADA2a (Figure 3C). The results indicated that the interaction between α-syn A53T and TADA2a was much stronger than that between α-syn WT and TADA2a and was accompanied by increased nuclear localization of α-syn A53T.

To confirm the interaction between α-syn A53T and TADA2a, GFP-tagged TADA2a and Flag-tagged α-syn WT or A53T were co-transfected into SH-SY5Y cells and their localizations were examined via immunofluorescence (Figure 3D). The results showed that TADA2a was predominantly localized in the nucleus, and a stronger signal of α-syn A53T compared with α-syn WT overlapped with TADA2a in the nucleus.

### 2.4. α-Syn A53T Inhibits Histone H3 Acetylation by Blocking TADA2a

**Figure 4:**
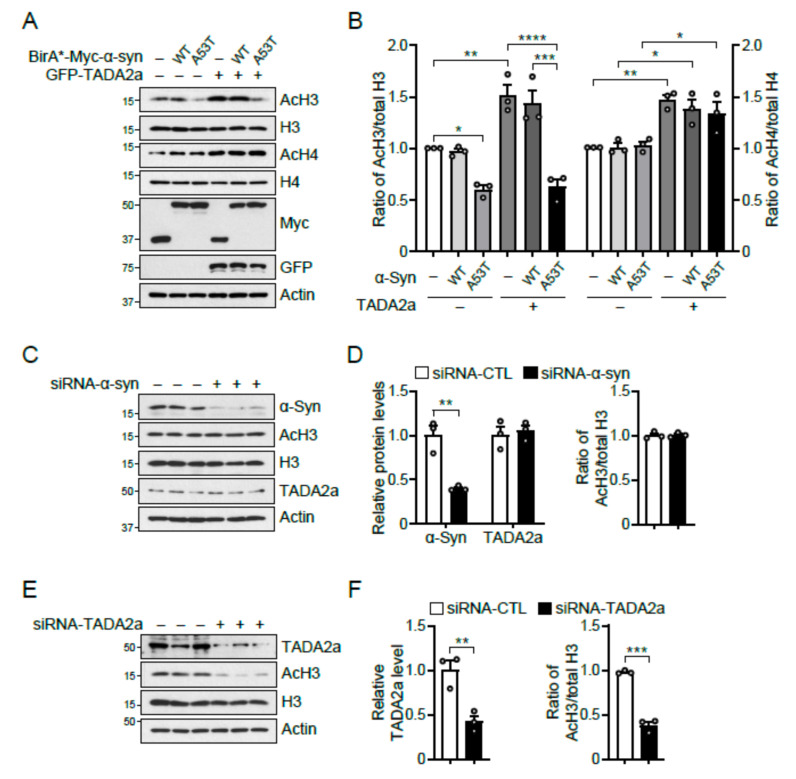
α-Syn A53T inhibits histone H3 acetylation by blocking TADA2a. (**A**) Immunoblot analysis of AcH3, histone H3, AcH4, histone H4, Myc, and GFP in SH-SY5Y cells transfected with BirA*-fused, Myc-tagged α-syn WT and A53T, and GFP-tagged TADA2a normalized to β-actin. (**B**) Quantification of the ratio of AcH3/total H3 and AcH4/total H4 from immunoblot analysis (A), *n* = 3 per group. (**C**) Immunoblot analysis of α-syn, AcH3, histone H3, and TADA2a in siRNA-α-syn knockdown SH-SY5Y cells normalized to β-actin. (**D**) Quantification of the levels of α-syn and TADA2a normalized to β-actin and the ratio of AcH3/total H3 from immunoblot analysis (C), *n* = 3 per group. (**E**) Immunoblot analysis of TADA2a, AcH3, and histone H3 in siRNA-TADA2a knockdown SH-SY5Y cells normalized to β-actin. (**F**) Quantification of the levels of TADA2a normalized to β-actin and the ratio of AcH3/total H3 from immunoblot analysis (E), *n* = 3 per group. Data = mean ± SEM. Statistical significance was determined by an unpaired, 2-tailed Student’s *t*-test (D and F) and ANOVA test with Tukey post hoc analysis (B). Differences were considered significant at *p* < 0.05. * *p* < 0.05, ** *p* < 0.01, *** *p* < 0.001, and **** *p* < 0.0001.

To test whether α-syn A53T affects the function of TADA2a, the levels of acetylated histone H3 (AcH3) were monitored in SH-SY5Y cells transfected with BirA*-fused, Myc-tagged α-syn WT or A53T, and GFP-tagged TADA2a (Figure 4A,B). TADA2a overexpression significantly upregulated the levels of AcH3, and α-syn A53T overexpression significantly suppressed the levels of AcH3 during the overexpression of either control or TADA2a (Figure 4A,B). However, the level of AcH3 was unchanged during the overexpression of α-syn WT, suggesting that the minimal localization and interaction between α-syn WT and TADA2a (Figure 3C,D) were insufficient to inhibit the role of TADA2a.

Since TADA2a is a subunit of p300/CBP-associated factor (PCAF), which is associated with histone H3 and histone H4 acetylation [17], the levels of acetylated histone H4 (AcH4) were also monitored in the same settings (Figure 4A,B). TADA2a overexpression increased AcH4 levels, whereas α-syn A53T overexpression did not affect the amount of AcH4, demonstrating that the mechanism underlying histone H3 and H4 acetylation by TADA2a differed to some extent and that α-syn A53T was involved in the process of TADA2a-mediated histone H3 acetylation.

To determine whether endogenous α-syn intrinsically participates in the regulation of histone H3 acetylation, AcH3 and TADA2a levels were measured in SH-SY5Y cells transfected with siRNA-α-syn. The results showed that there were no changes in the levels of AcH3 and TADA2a (Figure 4C,D). However, when endogenous TADA2a was knocked down using siRNA-TADA2a, AcH3 levels decreased, demonstrating that the expression of AcH3 depends on endogenous TADA2a and that TADA2a inhibition was achieved by the overexpression of α-syn, but not by endogenous α-syn alone (Figure 4E,F).

### 2.5. Reduction in TADA2a Levels and Histone H3 Acetylation in Intrastriatally α-Syn PFF-Injected Mice and the SN of Patients with PD

**Figure 5:**
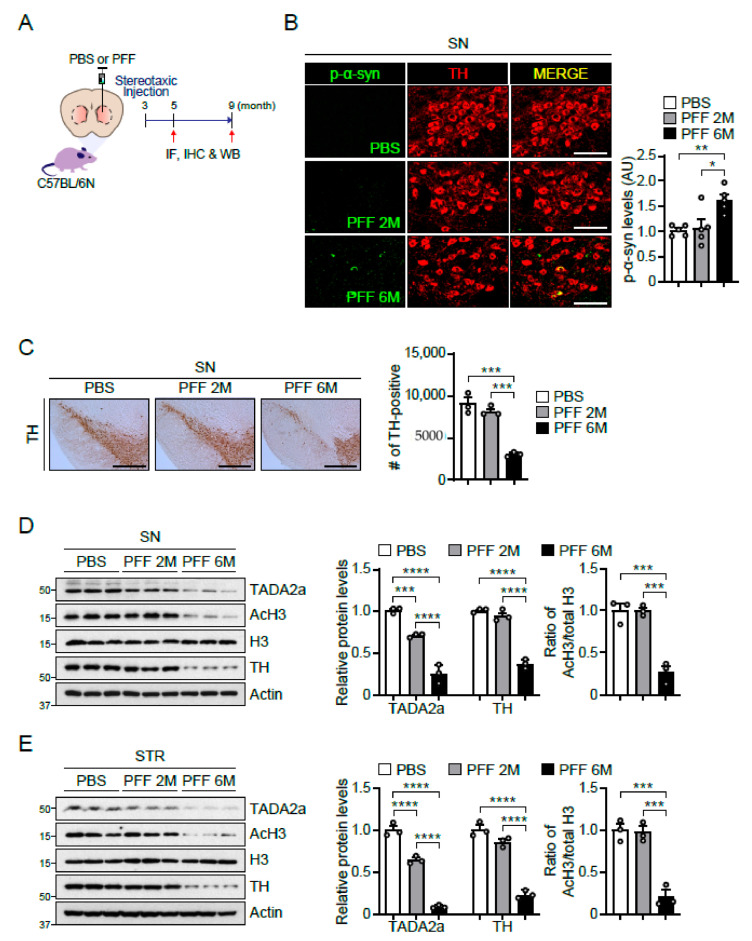
Reduction in TADA2a levels and histone H3 acetylation in intrastriatally α-syn PFF-injected mice. (**A**) Experimental scheme for generating α-syn PFF-injected mice. α-Syn PFF-injected mice were sacrificed for immunofluorescence (IF), immunohistochemistry (IHC), and immunoblot analysis (WB) at 2 and 6 months after injection. (**B**) Representative images of phosphorylated α-syn (p-α-syn, green) and TH (red) immunofluorescence in the SN of PBS- and α-syn PFF-injected mice (2 and 6 months post-injection; 2M and 6M). Scale bars = 100 μm. Quantification of p-α-syn immunofluorescence in the SN of indicated mouse groups, *n* = 5 per group (right panel). (**C**) Representative images of immunohistochemistry of TH in the SN of PBS- and α-syn PFF-injected mice. Scale bars = 500 μm. Stereological assessment of TH-positive dopaminergic neurons in the SN of indicated mouse groups, *n* = 3 per group (right panel). (**D**) Immunoblot analysis of TADA2a, AcH3, histone H3, and TH in the SN of PBS- and α-syn PFF-injected mice normalized to β-actin. Quantification of the levels of TADA2a and TH normalized to β-actin (middle panel) and the ratio of AcH3/total H3 (right panel), *n* = 3 per group. (**E**) Immunoblot analysis of TADA2a, AcH3, histone H3, and TH in the STR of PBS- and α-syn PFF-injected mice normalized to β-actin. Quantification of the levels of TADA2a and TH normalized to β-actin (middle panel) and the ratio of AcH3/total H3 (right panel), *n* = 3 per group. Data = mean ± SEM. Statistical significance was determined using ANOVA test with Tukey post hoc analysis (B–E). Differences were considered significant when *p* < 0.05. * *p* < 0.05, ** *p* < 0.01, *** *p* < 0.001, and **** *p* < 0.0001.

To validate the pathological relevance of AcH3 reduction, we utilized a well-characterized sporadic PD mouse model that was intrastriatally injected with α-syn preformed fibril (PFF). At post-α-syn PFF injection as indicated (Figure 5A), the retrograde transmission of α-syn PFF from the striatum (STR) into the SN was examined by immunostaining, confirming co-localization of phosphorylated α-syn (p-α-syn) and tyrosine hydroxylase (TH) in the SN at 6 months post-α-syn PFF injection (Figure 5B). Moreover, the loss of dopaminergic neurons by α-syn PFF injection was monitored via the immunohistochemistry of TH in the SN of α-syn PFF-injected mice (Figure 5C). Loss of dopaminergic neurons was observed at 6 months post-α-syn PFF injection but not at 2 months post-injection, accompanying the lack of p-α-syn signal in the SN (middle panel, Figure 5B). Next, the levels of TADA2a, AcH3, histone H3, and TH proteins were measured in the SN and STR (Figure 5D,E). Under these conditions, we also observed a significant reduction in TADA2a, AcH3, and TH in the SN at 6 months post-α-syn PFF injection (Figure 5D). A similar expression pattern was observed in the STR of α-syn PFF-injected mice (Figure 5E). These results indicated that α-syn PFF reduced the expression of TADA2a, leading to a decrease in AcH3 levels and dopaminergic neurodegeneration. Interestingly, at 2 months post-α-syn PFF injection, the levels of AcH3 and TH were unchanged, whereas the levels of TADA2a were reduced by approximately 30% (Figure 5D,E), indicating that α-syn PFF affects the function of TADA2a at an early stage of neurodegeneration.

**Figure 6:**
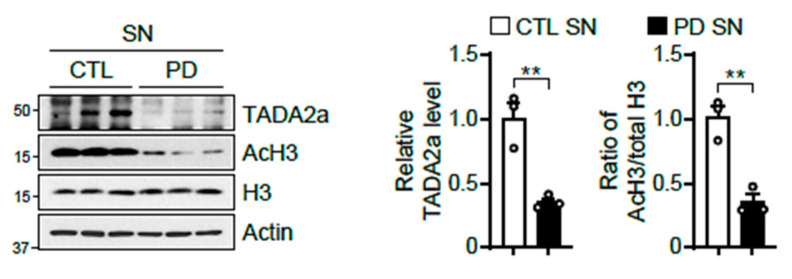
Reduction in TADA2a levels and histone H3 acetylation in the SN of patients with PD. Immunoblot analysis of TADA2a, AcH3, and histone H3 in the SN of the age-matched control (CTL) and patients with PD normalized to β-actin. Quantification of the level of TADA2a normalized to β-actin (middle panel) and the ratio of AcH3/total H3 (right panel), *n* = 3 per group. Data = mean ± SEM. Statistical significance was determined using an unpaired, 2-tailed Student’s *t*-test, ** *p* < 0.01.

Finally, we monitored whether these phenomena also occur in the post-mortem brains of patients with PD. Interestingly, the expression levels of TADA2a and AcH3 were lower in the SN of patients with PD (Figure 6), suggesting that dysregulation of AcH3 by malfunctional TADA2a can be a pathological event in synucleinopathies.

## 3. Discussion

The BioID system has been used as a tool for identifying candidate protein–protein interactions in living cells [18]. BioID helps to detect proximate proteins of the protein of interest, which is biotin ligase-fused [13]. This system also detects weak or transient interactions that occur in vivo and is appropriate for insoluble proteins without much influence on protein solubility. Owing to these advantages, BioID is used to complement existing methods for detecting protein–protein interactions such as yeast-2-hybrid or affinity-complex purification.

α-Syn is a highly conserved, 140 amino acid protein that is expressed in different regions of the brain [19]. Although α-syn does not have NLS, nuclear α-syn has been continuously studied [7,8,9,10,11,12]. For example, translocation of α-syn into the nucleus was observed in the brain of paraquat-exposed mice and H_2_O_2_-treated dopaminergic cells MES23.5 [8,20]. A recent study also found that sumoylated α-syn interacts with karyopherin α6, nuclear transport protein, facilitating nuclear translocation of α-syn [21]. In addition, importin α binds to α-syn and mediates the translocation of α-syn to the nucleus [22]. These results demonstrate that a variety of cellular stresses and α-syn’s interactors contribute to the nuclear entry of α-syn. In this study, we suppose that the overexpression of α-syn A53T increases ROS levels [23,24,25] and slightly weakens the integrity of the nuclear membrane [26] as compared to α-syn WT, explaining the stronger interaction of α-syn A53T with TADA2a.

Although α-syn is well-known for its synaptic function, little is known about its other roles [16]. Some researchers have suggested that α-syn can bind to DNA and alter the characteristics of both proteins and DNA [27]. Interestingly, α-syn can facilitate the non-homologous end-joining reaction and modulate the repair of DNA breaks [28]. In addition, some studies have demonstrated that α-syn and histones associate closely [8,9,29]. For example, a previous study reported that α-syn WT and A53T may bind directly to histones to regulate their acetylation. However, they failed to find a direct interaction between α-syn and HAT complex enzymes [9]. By identifying TADA2a as the proximal interactome of α-syn in this study, we provided clarification on how α-syn affects histone acetylation.

TADA2a has been studied as a key component of the PCAF and Ada two-A-containing (ATAC) HAT complexes [30] contributing to histone and non-histone acetylation. For example, TADA2a interacts with p53 to promote the acetylation of p53 at lysine 320, increasing its stability and activity [17,31]. TADA2a may also impact acetylation in a variety of ways during DNA damage-induced apoptosis and cell-cycle arrest [17]. In this study, we showed that α-syn-TADA2a interaction inhibited the acetylation of histone H3 but not of histone H4 (Figure 4A,B), even though both histone H3 and H4 are the substrates of TADA2a. It might result from the findings that α-syn binds directly to histone H3 [9], and TADA2a-containing PCAF is more effective on histone H3 acetylation [32]. Herein, our findings contribute to expanding knowledge of the role of TADA2a in α-syn-mediated neurotoxicity.

Histone H3 acetylation is considered to be very important for dopaminergic neuronal survival [8,9]. Inhibition of histone deacetylase (HDAC) activity is shown to be effective at reducing neurotoxicity and enhancing neuroprotection [9,33,34]. HDAC inhibitors have been used in a variety of animal models of diseases such as Huntington’s disease, amyotrophic lateral sclerosis (ALS), and spinal and bulbar muscular atrophy (SBMA) [35,36,37,38,39,40,41]. For example, sodium butyrate and suberoylanilide hydroxamic acid (SAHA), well-known HDAC inhibitors, reportedly rescued α-syn-associated neurodegeneration in SH-SY5Y cells and flies [9]. Therefore, maintaining the physiological level of histone H3 acetylation through the normal operation of the HAT complex and diverse HDAC inhibitors can be a novel therapeutic target for neurodegenerative diseases, including PD.

As mentioned, there are a number of studies demonstrating the role of α-syn in the nucleus [7,8,9,10,11,12]. Although controversy persists over the function of nuclear α-syn, our research has clearly shown that α-syn A53T preferentially exists in the nucleus and blocks TADA2a function. In the PD model, α-syn PFF led to dopaminergic neuronal loss and decreases in TADA2a and AcH3 levels in the mouse SN and STR. Furthermore, these molecular alterations were observed in the SN of the brains of patients with PD, suggesting that TADA2a is involved in the downregulation of AcH3 and neurodegeneration. Indeed, we observed a slight decline of TADA2a in the SN and STR of α-syn PFF-injected mice compared to PBS-injected mice at 2 months post-injection, whereas there were no changes in AcH3 and TH levels between groups (Figure 5D,E). Since the loss of dopaminergic neurons was not observed in the SN and STR of 2-months post-α-syn PFF-injected mice, we assume that the decrease of TADA2a may be prior to the AcH3 downregulation and the dopaminergic neurodegeneration. Further studies are required to understand how α-syn PFF reduces TADA2a levels in the SN and whether restoration of AcH3 can effectively prevent α-syn PFF-mediated neurotoxicity. In addition, the identification of target genes that are suppressed by reducing AcH3 levels in the PD model presents novel treatment options for synucleinopathy in PD.

## 4. Materials and Methods

### 4.1. Animals and Antibodies

All animal experiments were approved by the Sungkyunkwan University Ethical Committee in accordance with international guidelines (SKKUIACUC2020-11-13-1). Male C57BL/6N background mice were purchased from Orient (Suwon, Korea) and maintained under a 12 h dark/light cycle in air-controlled rooms with free access to diet and water. All efforts were made to reduce animal suffering and to minimize the number of animals used.

The following primary antibodies were used: rabbit anti-streptavidin (A00621-5, Genscript, Piscataway, NJ, USA), rabbit anti-myc (A190-105A, Bethyl Laboratories, Montgomery, TX, USA), mouse anti-GFP (sc-9996, Santa Cruz, Dallas, TX, USA), rabbit anti-fibrillarin (sc-25397, Santa Cruz, Dallas, TX, USA), rabbit anti-acetyl-histone H3 (Lys9/Lys14) (9677S, Cell Signaling, Danvers, MA, USA), rabbit anti-histone H3 (D1H2) (4499P, Cell Signaling, Danvers, MA, USA), rabbit anti-acetyl-histone H4 (Lys8) (2549T, Cell Signaling, Danvers, MA, USA), mouse anti-histone H4 (D2X4V) (13919S, Cell Signaling, Danvers, MA, USA), rabbit anti-α-syn (2642S, Cell Signaling, Danvers, MA, USA), mouse anti-p-α-syn (MMS-5091, BioLegend, San Diego, CA, USA), mouse anti-Flag (F1804, Sigma-Aldrich, St. Louis, MO, USA), rabbit anti-TH (NB300-109, Novus Biologicals, Centennial, CO, USA), mouse anti-Hsp90 (Ab13592, Abcam, Cambridge, UK), and rabbit anti-TADA2a (Ab222309, Abcam, Cambridge, UK).

HRP-conjugated sheep anti-mouse IgG (RPN4301, GE Healthcare, Chicago, IL, USA) and HRP-conjugated donkey anti-rabbit IgG (RPN4101, GE Healthcare, Chicago, IL, USA) were used as secondary antibodies for the immunoblotting assays. Donkey anti-mouse IgG H&L (Alexa Fluor 488) (Ab150105, Abcam, Cambridge, UK), donkey anti-rabbit IgG H&L (Alexa Fluor 594) (Ab150076, Abcam, Cambridge, UK), and biotin-SP-AffiniPure goat anti-rabbit IgG H+L (111-065-045, Jackson ImmunoResearch, Philadelphia, PA, USA) were used as secondary antibodies for the immunofluorescence and immunohistochemistry assays.

### 4.2. Plasmid Construction

The pcDNA3.1 mycBioID vector was purchased from Addgene (plasmid #35700). BirA*-fused, Myc-tagged α-syn WT and A53T were constructed by ligating full-length α-syn WT and A53T, respectively, into the XhoI (forward) and KpnI (reverse) restriction sites of the mycBioID vector and the sequences were confirmed using automated DNA sequencing. pcDNA6.2 emGFP α-syn WT/A53T, pEZY Flag α-syn WT/A53T, and pcDNA6.2 emGFP TADA2a were generated by recombining each expression vector with α-syn WT/A53T or TADA2a human cDNA using LR clonase II (Invitrogen, Carlsbad, CA, USA) and then cloning into Stbl3 *E. coli*-competent cells.

Human siRNA-CTL, -α-syn, and -TADA2a were purchased from Bioneer Inc. (Daejeon, Korea) for use in the analysis of the effect of knockdown of human TADA2a and α-syn in SH-SY5Y cells. RNA interference efficiency was confirmed through western blot analysis.

### 4.3. Cell Culture and Transfection

Human neuroblastoma SH-SY5Y cells (ATCC, Manassas, VA, USA) were grown in DMEM (WELGENE, Gyeongsan, Korea), containing 10% FBS (*v*/*v*, WELGENE, Gyeongsan, Korea) and antibiotics (penicillin/streptomycin 100 U/100 μg per mL, Gibco, Carlsbad, CA, USA), in a humidified 5% CO_2_/95% air atmosphere at 37 °C (Forma Direct Heat CO_2_ incubator, Thermo Fisher Scientific, Waltham, MA, USA). X-tremeGENE HP transfection reagent (Roche, Basel, Switzerland) was used for transient transfections according to the manufacturer’s instructions. Unless otherwise indicated, lysates were prepared 48 h after transfection.

### 4.4. BioID Pulldown Assay

The BioID expression vector ligated with α-syn WT or A53T was transfected into SH-SY5Y cells. Biotinylation was induced by treatment with 50 μM biotin. Cells were washed with ice-cold PBS and harvested in the lysis buffer (50 mM Tris-HCl, pH 7.5, 500 mM NaCl, 2 mM EDTA, 1 mM DTT, 0.4% (*w*/*v*) SDS, and a protease inhibitor cocktail (GenDEPOT, Barker, TX, USA)) 24 h after biotinylation. After sonication, the lysates were then centrifuged at 22,250× *g* for 20 min. Biotinylated proteins in the supernatants were captured on streptavidin beads by rotation overnight at 4 °C. The following day, the beads were pelleted and washed thrice with wash buffer A (50 mM HEPES, 500 mM NaCl, 1% (*v*/*v*) Triton X-100, 1 mM EDTA, and a protease inhibitor cocktail (GenDEPOT, Barker, TX, USA)) and twice with wash buffer B (50 mM Tris-HCl, pH 7.5, 50 mM NaCl, and a protease inhibitor cocktail (GenDEPOT, Barker, TX, USA)). Beads were then mixed with 2× SDS sample buffer (Bio-Rad, Hercules, CA, USA) and boiled at 95 °C for 15 min. The reaction was verified via streptavidin immunoblot analysis; coomassie-stained gel was used, in combination with mass spectrometry, to identify the proteins.

### 4.5. Mass Spectrometric Analysis

Mass spectrometry was performed as previously reported [42]. Briefly, gel pieces were de-stained, reduced, alkylated, and digested with modified sequencing-grade trypsin (Promega, Madison, WI, USA). Peptide pools were resuspended in 0.1% TFA and applied into a Zorbox 300SB-C18 75 μm i.d. × 15 cm column (Agilent, Waldbronn, Germany) via a trap column (Zorbox 300SB-C18 300 μm i.d. × 5 mm column, Agilent, Waldbronn, Germany). The collected peptides were further separated using an acetonitrile gradient (buffer A: 0.1% formic acid; buffer B: 100% acetonitrile and 0.1% formic acid) at a flow rate of 200 nL/min with an UltiMate 3000 HPLC system (Dionex, Sunnyvale, CA, USA) and applied online to an LTQ ion-trap mass spectrometer (Thermo Fisher Scientific, Waltham, MA, USA). The gradient was increased from 5% to 40% buffer B for 110 min, then increased to 80% B for 1 min, and finally to 80% B isocratic for 15 min. Full scan mode was utilized for MS spectra acquisition (350–1600 Da), followed by MS/MS scans with the 5 most intense ions.

### 4.6. Quantitative Protein Profiling and Database Searching

Quantitative protein profiling and database searches were completed as previously mentioned [43]. Briefly, MS/MS spectra corresponding to peptide peaks were applied to the Uniprot database using Mascot™ 2.3 (Matrix Science, London, UK). The search parameters were set as follows: enzyme, trypsin; missed cleavage sites allowed, 3; fixed modification, carbamidomethyl; variable modifications, oxidation of methionine; precursor mass tolerance, 2 Da; fragment mass tolerance, 1 Da. The peptide identifications were subject to DeCyder MS software. For quantitative profiling, we selected proteins that were identified by multiple peptides with a significant Mascot score (*p* < 0.05) [44]. To validate MS/MS-based peptide and protein identifications, we used the Scaffold 3.06 software (Proteome Software Inc., Portland, OR, USA) and MS/MS samples were analyzed using Mascot™ 2.3 (Matrix Science, London, UK). The parameters used were as follows: peptide identification probability of >95%, protein identification probability of > 99%, and a minimum of 2 peptides.

### 4.7. In Silico Analysis of Functional Associations

In silico analysis of functional associations was performed as previously reported [45]. Briefly, we used STRAP (http://www.bumc.bu.edu/cardiovascularproteomics/cpctools/, accessed on 1 April 2021, Boston University School of Medicine, Boston, MA, USA) to classify proteins according to the criteria biological process, cellular component, and molecular function based on gene ontology (GO). We also used a STRING 8.3 webserver (http://string-db.org/, accessed on 1 April 2021) to investigate the functional association between interactors.

### 4.8. Subcellular Fractionation

SH-SY5Y cells transfected with either GFP-tagged α-syn WT or A53T were subjected to subcellular fractionation. Harvested cells were mixed with buffer A (0.01 M HEPES, 10 mM KCl, 1.5 mM MgCl_2_, 1 mM EDTA, 1 mM EGTA, 1 mM DTT, and 0.075% (*v*/*v*) NP40), containing a protease inhibitor cocktail (GenDEPOT, Barker, TX, USA), and incubated for 10 min on a rotator. After centrifugation at 750× *g* for 10 min, the supernatants containing cytoplasmic proteins were transferred to new tubes placed on ice. The remaining pellets were suspended in buffer B (0.01 M HEPES, 10 mM KCl, 1.5 mM MgCl_2_, 1 mM EDTA, 1 mM EGTA, 1 mM DTT, 0.075% (*v*/*v*) NP40, and 0.25 M sucrose), containing a protease inhibitor cocktail (GenDEPOT, Barker, TX, USA), and incubated on a rotator for 20 min, followed by centrifugation at 750× *g* for 10 min. After removing the supernatants, the pellets were suspended in buffer A to dissolve the nuclear proteins.

### 4.9. Immunocytochemistry

Cells transfected with Flag-tagged α-syn WT/A53T and GFP-tagged TADA2a were fixed with ice-cold 4% (*w*/*v*) paraformaldehyde. For blocking and permeabilization, the cells were incubated in 10% (*v*/*v*) goat serum/0.1% (*v*/*v*) Triton X-100 for 30 min at 25 °C. Primary antibodies were diluted with a blocking buffer. Cells were probed with the diluted primary antibodies overnight at 4 °C and then incubated with the appropriate fluorescence-conjugated secondary antibodies at 25 °C for 1 h. Images were acquired using a confocal microscope (Leica, Wetzlar, Germany).

### 4.10. Co-Immunoprecipitation

For the co-immunoprecipitation experiments, SH-SY5Y cells were washed with ice-cold PBS and harvested in 1% (*v*/*v*) Triton X-100 (in PBS) 48 h after transfection. The lysates were rotated at 4 °C for 1 h and then centrifuged at 22,250× *g* for 20 min. The supernatants were combined with protein G Sepharose beads (Amersham Biosciences, Piscataway, NJ, USA), pre-incubated with the antibody against Myc (A190-105A, Bethyl Laboratories, Montgomery, TX, USA), and mixed by rotation overnight at 4 °C. The following day, protein G Sepharose beads (Amersham Biosciences, Piscataway, NJ, USA) were pelleted and washed thrice with 1% (*v*/*v*) Triton X-100. The precipitates were resolved using SDS-PAGE and subjected to immunoblot analysis.

### 4.11. Histone Extraction for Histone Acetylation Analysis

Cells were harvested and washed twice with ice-cold PBS. PBS and subsequent buffers were supplemented with 5 mM sodium butyrate to maintain the histone acetylation levels. Cells were resuspended in Triton extraction buffer (PBS with 0.5% (*v*/*v*) Triton X-100, 2 mM phenylmethylsulfonyl fluoride (PMSF), and 0.02% (*w*/*v*) NaN_3_), at a density of 10^7^ cells/mL and lysed on ice for 10 min with gentle stirring. The lysates were then centrifuged at 6500× *g* for 10 min at 4 °C to spin down the nuclei. After removing the supernatants, pellets were resuspended in 0.2 N HCl at a density of 4 × 10^7^ nuclei/mL overnight at 4 °C. The following day, samples were rotated at 6500× *g* for 10 min at 4 °C to pellet the debris. Supernatants containing histone proteins were retained and neutralized with 2 M NaOH at 1/10 volume of the supernatant. After determining the protein content using the Bradford assay, the samples were mixed with 2× SDS sample buffer (Bio-Rad, Hercules, CA, USA) and boiled at 95 °C for 15 min.

### 4.12. Stereotaxic Injection of α-Syn PFF

α-Syn PFF was prepared as previously described [46]. Three-month-old male C57BL/6N mice (Orient, Suwon, Korea) were anesthetized with isoflurane. Approximately 2 μL of α-syn PFF (total 5 μg) or PBS was injected into the STR (0.5 μL/min) using the following coordinates: anteroposterior = +0.5 mm, mediolateral = ±2 mm, and dorsoventral = −3 mm from bregma. The needle was maintained for an additional 3 min to allow complete elution of the solution. After injection, all animals were subjected to post-surgical care. After 2 and 6 months of injection, the mice were euthanized for further studies.

### 4.13. Immunohistochemistry and Stereological TH-Positive Neuron Assessment

Mice were anesthetized with isoflurane and perfused with ice-cold PBS. Brains were fixed with ice-cold 4% (*w*/*v*) paraformaldehyde overnight and cryoprotected in 30% (*w*/*v*) sucrose (in PBS) overnight at 4 °C. Thirty-five μm coronal sections were probed with the primary antibodies in the blocking buffer (PBS with 10% (*v*/*v*) goat serum and 0.1% (*v*/*v*) Triton X-100) overnight at 4 °C and then incubated with the appropriate fluorescence-conjugated secondary antibodies at 25 °C for 1 h. Images were acquired using a confocal microscope (Leica, Wetzlar, Germany).

For TH counting, sections were probed with rabbit anti-TH (NB300-109, Novus Biologicals, Centennial, CO, USA) in the blocking buffer overnight at 4 °C and then incubated with biotin-SP-AffiniPure goat anti-rabbit IgG H+L (111-065-045, Jackson ImmunoResearch, Philadelphia, PA, USA) at 25 °C for 1 h. We used streptavidin-conjugated HRP (Vectastain ABC kit, PK4000, Vector Laboratories, Burlingame, CA, USA) and 3,3′-diaminobenzidine tablets (D4293, Sigma-Aldrich, St. Louis, MO, USA) to visualize TH-positive neurons. The counting of total numbers of TH-positive neurons in the SN was conducted via an Optical Fractionator probe of Stereo Investigator software (MicroBrightfield, Williston, VT, USA).

### 4.14. Preparation of Brain Tissues from Mice and Patients with PD for Immunoblot Assay

Human and mice brain tissues were harvested and homogenized in RIPA lysis buffer (50 mM Tris-HCl, pH 7.5, 150 mM NaCl, 2 mM EDTA, 1% (*v*/*v*) Triton X-100, 0.5% (*w*/*v*) sodium deoxycholate, 0.1% (*w*/*v*) SDS, phosphatase inhibitor cocktail I and II (Sigma-Aldrich, St. Louis, MO, USA), and a protease inhibitor cocktail (GenDEPOT, Barker, TX, USA) using a Diax 900 homogenizer. After homogenization, the samples were rotated at 4 °C for 30 min to ensure complete lysis. The lysates were mixed with 2× SDS sample buffer (Bio-Rad, Hercules, CA, USA) and then boiled at 95 °C for 15 min. Protein concentrations were quantified using a bicinchoninic acid kit (Pierce, Waltham, MA, USA) with BSA standards. Immunoblotting was performed with the antibodies of interest and visualized using chemiluminescence (Pierce, Waltham, MA, USA). Densitometric analysis of the immunoblot bands was performed using ImageJ software (NIH, http://rsb.info.nih.gov/ij/, downloaded on 2 March 2017). Information on the PD specimens used in this study has been described previously [43].

### 4.15. Quantification and Statistical Analysis

Quantitative data are presented as mean ± standard error of the mean (SEM). Statistical significance was assessed either via an unpaired, 2-tailed Student’s *t*-test for comparisons of 2 groups or an ANOVA test with Tukey’s HSD post hoc analysis for comparisons of more than 3 groups. Assessments were considered significant at * *p* < 0.05, ** *p* < 0.01, *** *p* < 0.001, and **** *p* < 0.0001.

## 5. Conclusions

In this study, we identified TADA2a as a novel interactome of α-syn A53T and found that α-syn A53T disturbed the function of TADA2a, leading to a reduction in histone H3 acetylation. In addition, the downregulation of TADA2a and histone H3 acetylation were observed in the SN and STR of α-syn PFF-injected mice and the SN of the brains of patients with PD, respectively. These findings may shed light on how the dysregulation of chromatin structure is associated with PD pathogenesis.

## Data Availability

The data presented in this study are available within the text and figures. Details are available on request from the corresponding author.

## Supplementary Materials

The following are available online at https://www.mdpi.com/article/10.3390/ijms22105392/s1, Table S1: Mass spectrometric identification of immunoprecipitates from the BioID pulldown assay.

## Abbreviations

PD	Parkinson’s disease
SN	substantia nigra
α-syn	α-synuclein
ROS	reactive oxygen species
NLS	nuclear localization sequence
TADA2a	transcriptional adapter 2-alpha
HAT	histone acetyltransferase
AcH3	acetylated histone H3
PCAF	p300/CBP-associated factor
AcH4	acetylated histone H4
PFF	preformed fibril
STR	striatum
p-α-syn	phosphorylated α-syn
TH	tyrosine hydroxylase
ATAC	Ada two-A-containing
HDAC	histone deacetylase
ALS	amyotrophic lateral sclerosis
SBMA	spinal and bulbar muscular atrophy
SAHA	suberoylanilide hydroxamic acid
